# Failure Prediction in 3D Printed Kevlar/Glass Fiber-Reinforced Nylon Structures with a Hole and Different Fiber Orientations

**DOI:** 10.3390/polym14204464

**Published:** 2022-10-21

**Authors:** Mohammed Aqeel Albadrani

**Affiliations:** Department of Mechanical Engineering, College of Engineering, Qassim University, Unaizah 56452, Saudi Arabia; moa.albadrani@qu.edu.sa

**Keywords:** finite element analysis, additive manufacturing, fiber reinforcement, 3D printing, Kevlar, glass fiber, nylon

## Abstract

This study examined the mechanical performance of 3D-printed, fiber-reinforced composites with a rectangular shape and a hole at one end. Nyon-6 was selected as a polymer matrix, and glass or Kevlar fibers were selected as continuous fibers due to their wide range of applications. Nylon is an engineering thermoplastic; reinforcing it with fibers, such as glass fiber or Kevlar, can significantly improve its mechanical properties. An analytical model was constructed based on the volume average stiffness approach to predict the mechanical properties of 3D-printed specimens. A numerical model was built to predict failure modes and damage in 3D-printed specimens with different fiber orientations. The stress–strain relationship was linear in all composites. For Kevlar-based composites, the maximum stress was 1.7 MPa, 3.62 MPa, 2.2 MPa, 1.0 MPa, and 1.4 MPa for the orientation angles of 0°, 22.5°, 45°, 67.5°, and 90°, respectively. Overall, Kevlar-based composites exhibited mechanical properties superior to those of glass-based composites. The effect of the fiber orientation was also different between the two systems. The simulation results predicted that the failure propagation begins in the areas close to the hole. Notably, the level of agreement between the simulated and experimental results varied depending on the fiber type and orientation, reflecting the complex interplay between multiple fibers, matrix interactions, and stress transfer.

## 1. Introduction

Fused deposition modeling (FDM) is an important approach in additive manufacturing (3D printing) that can be widely used to create novel structures [1,2,3]. The 3D structures are formed by feeding heated filaments into an extruder that melts them, and precise printing pathways, along with printing parameters, are then built and programmed to control the movement of the extruder nozzle to create a certain shape [4]. The structure is created layer by layer as the deposited material accumulates. However, most 3D-printable thermoplastics have poor mechanical properties, which are unfavorable for outdoor applications, structural objects, sports gears, and other objects that experience dynamic and static loads, making it necessary to develop strategies to further improve the mechanical properties of 3D-printed objects.

Polymer fiber composites are developed by mixing reinforcing fibers with a polymer matrix [5,6]. Fiber-based fillers have a high stiffness-to-weight ratio and are often utilized as reinforcing materials [7,8,9,10]. Fiber-reinforced 3D printing is an emerging additive manufacturing method with improved production and prototyping capabilities. It involves the simultaneous extrusion of fiber and thermoplastics to create superior artifacts; however, these artifacts are vulnerable to structural stresses, and it is important to develop accurate predictive models and numerical simulations of mechanical failure. The reinforcing fibers used in these composites can be manufactured from a wide range of materials, such as glass, carbon, and Kevlar [11]. Owing to their excellent strength-to-weight ratio, polymer fiber composites are widely used in the aerospace and automotive sectors. They have also been explored in the building sector, where they provide substitutes for steel or concrete in a variety of applications. Nylon is soft and easy to deform when heated above the melting temperature, making it among the most widely used engineering thermoplastics in 3D printing [12,13]. Nylon-based polymer composite materials have controlled combinations of properties that are achievable using fused filament fabrication technology in 3D printing. Nylon plays a vital role in many sectors due to its mechanical properties and lightweight nature [14]; for example, in the automotive sector, nylon composites are often used when high strength and low weight are desired.

3D printers with continuous fiber reinforcement have been designed to maintain the advantages of flexibility and ease of processing of 3D printing while improving their mechanical performance [15,16]. Because the matrix is thermoplastic, these printers can simply 3D print structures along with the embedded fiber at a slightly higher extrusion temperature to reinforce the structure in the direction of fiber alignment. Using these advanced 3D printers, bilayer fiber-reinforced composites can be formed, which are relatively difficult to fabricate using traditional approaches.

Finite element analysis (FEA) is commonly used to predict the deformation of a designed structure [17]. It is a numerical approach for solving engineering and applied science problems in a broad range of domains, such as mechanics, electronics, electromagnetics, and aerospace engineering. In the field of polymer fiber composites, FEA is used to investigate the behavior of materials under various loading conditions, determine how the material will react to the applied loads, and identify the probable failure points [18]. FEA is a powerful tool for studying the behavior of polymer fiber composites under a variety of loading conditions. In addition, FEA can be used to optimize the design of stronger and more durable composite structures. Because FEA can play an important role in modeling the stress-induced failure of 3D-printed FRPs, considerable research has been conducted to simulate the properties of polymeric materials, including 3D-printing filament materials. Kalova et al. examined a continuous carbon fiber-reinforced onyx matrix, that is, a matrix consisting of nylon and microcarbon fibers. FEM analysis revealed that the primary failure of 3D-printed composite parts was not due to loss of stability but to material failure [19]. Calignano et al. analyzed the actual mechanical characteristics of parts fabricated using carbon fiber-reinforced nylon filaments and reported that the obtained values differed considerably from the values presented in the datasheets of various filament suppliers. Furthermore, hardness and tensile strength are influenced by the direction of the printing, the percentage of filling, and thermal stresses, while resilience is affected only by the direction of the printing, and the relationship between mechanical properties and the filling factor is not linear [20]. In another study, a progressive damage approach was used to simulate the mechanical responses of carbon, Kevlar, and glass fiber-reinforced nylon composites (40% fiber volume fraction) using the Abaqus [21]. The quantity of reinforcing fibers and the fiber orientation were discovered to have a substantial influence on the structural integrity of the printed composites.

The above-mentioned studies reflect the potential of FEA-based methods in predicting the properties of 3D-printed FRPs; however, the geometries investigated were simple, and fiber orientation was investigated only at a limited number of angles, reflecting the need for more extensive studies to establish the feasibility of FEA-based simulations in predicting the mechanical properties. Furthermore, high-quality holes are needed for several applications, such as for rivets and fastening components; however, the drilling process can adversely affect the mechanical characteristics of the object [22]. It is, therefore, critical to investigate the mechanical performance of 3D-printed objects with holes; however, such aspects have received little attention.

This paper describes the fabrication of 3D-printed, fiber-reinforced structures with multiple orientations and a circular hole in their geometry, as well as their mechanical characterization and FEA. The analytical approach adopted was based on the volume-averaging stiffness (VAS) method [15,16,23]. The failure prediction approach relied on recording damage parameters in different failure modes by considering matrix and fiber damages under compressive and tensile stresses. The remainder of this paper is organized as follows. Section 2 briefly introduces the geometric scope, structural design, and fabrication parameters. Section 3 mainly discusses the physics behind the material properties and precise material characterization and introduces simulation modeling and sequential FEA. Section 3 shows some simulations and design results derived from the proposed model. Finally, Section 4 concludes the study.

## 2. Experimental and Analytical Studies

### 2.1. Materials

Kevlar or glass fiber was used as the filler, and nylon 6 was used as the matrix. Nylon 6 is a polyamide with a melting temperature of 210 °C. The continuous filament fabrication process was used, and the densities of the Kevlar, glass fiber, and nylon 6 were 1.2 g/cc, 1.5 g/cc, and 1.1 g/cc, respectively.

### 2.2. 3D Printing

**Figure 1:**
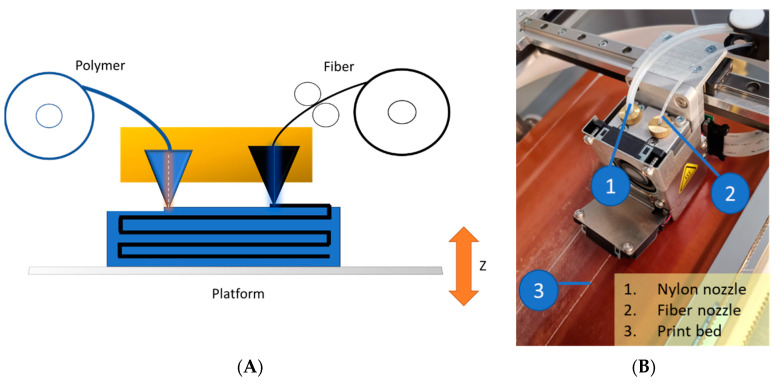
(**A**) Schematic representation of the 3D printing process for continuous fiber-reinforced polymer composites and (**B**) a photograph of the 3D printer used in the study.

**Figure 2:**
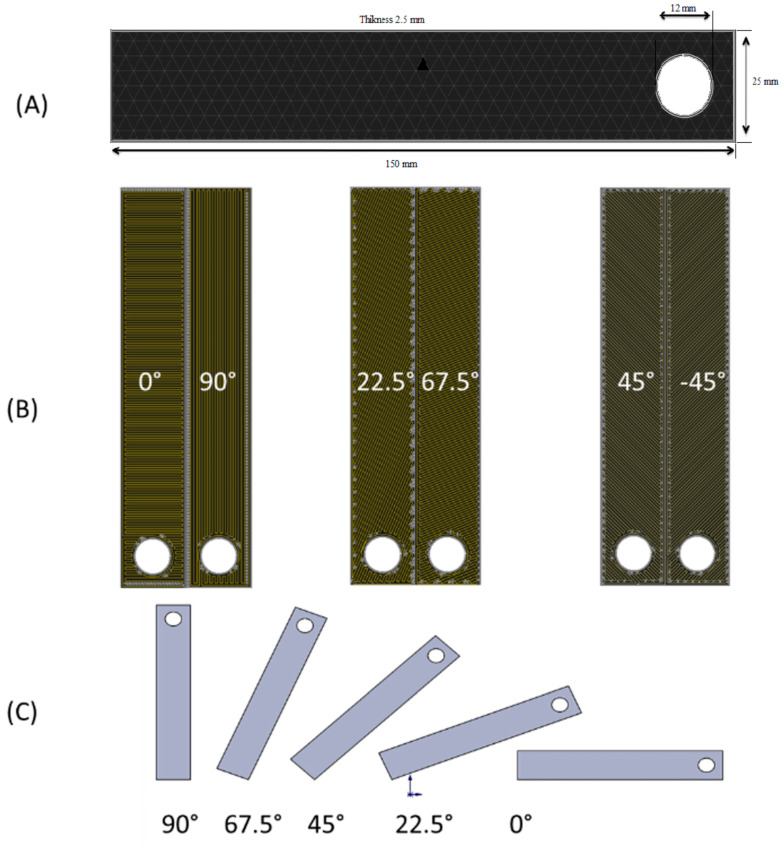
(**A**) The 3D-printed, fiber-reinforced structure, (**B**) different fiber orientation configurations, and (**C**) CAD geometry according to different fiber orientation configurations.

The Mark Two 3D printer was used to develop continuous fiber-reinforced composites (MarkForged, Somerville, MA, USA) [24]. This printer has two distinct nozzles: one extruding nylon and the other providing Kevlar fiber (Figure 1). The fiber is printed first and then embedded in the polymer matrix. The diameter of the filament was 0.34–0.38 mm, and the specimen thickness was 2.5 mm. The temperature at which polyamide was applied to the fiber was 90 °C. As the mechanical characteristics of composites are affected by the distribution of fiber layers, tensile test coupons with various fiber orientations were fabricated. The stacking sequences of the tested specimens involved angles of 45°, −45°, 90°, and 0°. It should be mentioned that fiber layers with homogeneous distributions and optimal orientation enhance composites by equally spreading tensile stress throughout the composite [24]. The geometry of the 3D-printed, fiber-reinforced structure is shown in Figure 2. The object was a rectangular bar of 150 mm × 25 mm × 2.5 mm (L × W × H). All specimens were reinforced with Kevlar or glass fiber in different orientations (0–90°) with different layers (Figure 2A–C).

**Figure 3:**
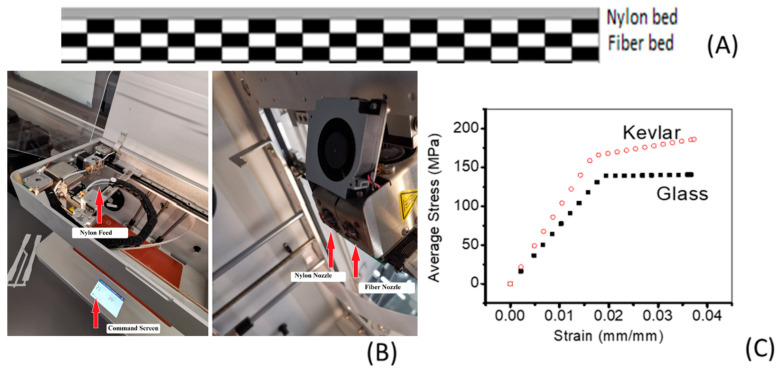
(**A**) Typical cross-section of the printed coupons, (**B**) 3D printer, and (**C**) stress–strain profiles generated using FEA for nylon/glass fiber and nylon/Kevlar composites.

Orientation angles of 45°, −45°, 90°, and 0°, and a 40% fiber volume fraction were selected. A typical cross-sectional profile of the printed coupons is shown in Figure 3A. In these coupons, five layers were printed (three layers of polymer and two layers of fiber). Important material properties are presented in Table 1.

### 2.3. Mechanical Properties

The tensile tests were performed using a universal testing machine operated at a constant loading rate of 2 mm/min. Mechanical tests were performed according to ASTM D3039 for composite laminates with differential fiber orientations and layers for both Kevlar and glass fibers.

### 2.4. Analytical Studies

A VAS-based analytical model, which assumes continuity of strain, was developed to predict the elastic properties of 3D-printed specimens. From a mechanical viewpoint, the continuity of strain is a reasonable assumption because errors are expected to be lower when strain is kept constant instead of stress due to the unevenness of the laminated composite section.

### 2.5. Finite Element Analysis

The software Abaqus was used to perform the finite element analysis. The element size used in the analysis was 2.5 mm. The specimen was constrained at all degrees of freedom on the bottom surface and all degrees of freedom, except for the loading direction, on the top surface. The sensitivity of element size was examined from 0.5 to 25 mm. At the tabbed ends, the boundary conditions of the model included the restraint of all transitional and rotational degrees of freedom, except for the axial movement. The reference point was selected at the center of the top edge, through which the axial tensile load was applied.

The first damage model, known as progressive failure analysis, was based on Hashin’s criteria to characterize the onset of damage. Damage initiation due to fiber tension (HSNFTCRT) or compression (HSNFCCRT) and matrix tension (HSNMTCRT) or compression (HSNMTCRT) are the four components of Hashin’s damage initiation criteria (HSNMCCRT). The abbreviations here correspond to Hashin (HSN), criteria (CRT), matrix (M), fiber (F), compressive (C), and tensile (T). The commencement of a specific component (by reaching a value of 1 within a certain parameter) and the potential of damage development were determined by the fulfillment of any stated component.

Tension failure criterion of the fiber:

(1) $$\frac{\sigma_{1}}{f_{1T}}=1$$

Compression failure criterion of the fiber:

(2) $$\frac{\sigma_{1}}{f_{1C}}=1$$

Tension failure criterion of the matrix:

(3) $$\left[\frac{{\left(\sigma_{2}+\sigma_{3}\right)}^{2}}{f_{2T}^{2}}+\frac{\tau_{23}^{2}-\sigma_{2}\sigma_{3}}{f_{2s}^{2}}+\frac{\tau_{31}^{2}-\tau_{12}^{2}}{f_{1s}^{2}}\right]=1$$

Compression failure criterion of the matrix:

(4) $${\left[\left[{\left(\frac{f_{2C}}{2f_{2s}}\right)}^{2}-1\right]\frac{\sigma_{2}+\sigma_{3}}{f_{2C}}+\frac{{\left(\sigma_{2}+\sigma_{3}\right)}^{2}}{4f_{2s}^{2}}+\frac{\left(\tau_{23}^{2}-\sigma_{2}\sigma_{3}\right)}{f_{2s}^{2}}+\frac{\left(\tau_{31}^{2}+\tau_{12}^{2}\right)}{f_{1s}^{2}}\right]}^{\frac{1}{2}}=1$$

where $f_{1T}$ is axial tension strength, $f_{1C}$ is axial compression strength, $f_{2T}$ is transverse tension strength, $f_{2C}$ is transverse compression strength, $f_{1s}$ is axial shear strength, $f_{2s}$ is transverse shear strength, and $\sigma_{i}$ and $\tau_{ij}$ (i and j = 1, 2, 3) are principal stresses. The damage parameters available at Abaqus are as follows: DAMAGEFT, fiber tensile damage; DAMAGEFC, fiber compression damage; DAMAGEMT, matrix tensile damage; DAMAGEMC, matrix compression damage.

## 3. Results and Discussion

The FEA-simulated stress–strain profiles for Kevlar and glass-reinforced nylon are compared in Figure 3C. The stress increased linearly with applied strain for both composites up to a strain of approximately 0.2. After that, the stress reached a plateau in the glass fiber-reinforced compound, and in Kevlar fiber-reinforced composites, it showed an incremental increase. The strain at break was approximately 4%, which is consistent with the values reported by Dikson et al. [25]. Nylon is an engineering thermoplastic; reinforcing it with fibers, such as glass fiber or Kevlar, can significantly improve its mechanical properties [26,27]. Most importantly, the tensile stress generated in the Kevlar composites was higher than that in the glass fiber composites, reflecting the higher stiffness of the Kevlar composites. This deviation can be traced to the fundamental differences between the properties of the material and the micromechanics of the system. Kevlar has longitudinal and transverse elastic moduli of 30,000 MPa and 10,000 MPa, respectively, whereas glass fibers have longitudinal and transverse elastic moduli of 25,000 MPa and 5000 MPa, respectively. The in-plane shear modulus is considerably higher in the case of glass fiber. However, since the current analysis was performed in a uniaxial tensile mode, it is expected that Kevlar composites will have superior mechanical properties. Other factors that contribute to differences include the impact of fibers on the transcrystalline zone [28] and interfacial interactions [29]. Notably, a previous study reported a higher reinforcing effect in nylon/glass fiber composites than in nylon/Kevlar composites [25]. These differences suggest that the fiber orientation and loading may have an influence, as detailed below.

**Figure 4:**
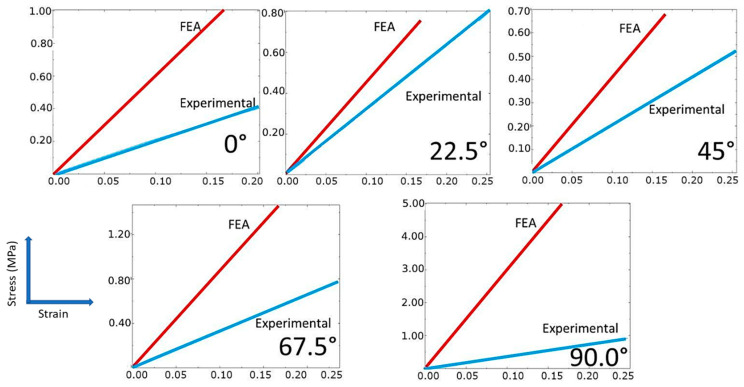
Stress–strain response of nylon/glass fiber composites with different orientations.

Not only the fundamental material properties of the fiber but also the orientation of the fibers affect the mechanical properties [30,31]. For different orientation angles, the stress–strain profiles (experimental and simulated) for glass fiber composites are shown in Figure 4.

The maximum experimental stress was 0.40 MPa, 0.80 MPa, 0.52 MPa, 0.77 MPa, and 0.92 MPa for the orientation angles of 0°, 22.5°, 45°, 67.5°, and 90°, respectively, and the maximum strain was 0.20, 0.25, 0.25, 0.24, and 0.25 for the corresponding orientation angles. The deviation between the experimental and simulated profiles varied depending on the orientation angle. The deviation was highest at 90° and lowest at 22.5°. In the simulated profiles, the maximum stress was 0.99 MPa, 0.75 MPa, 0.68 MPa, 1.5 MPa, and 5.0 MPa for the orientation angles of 0°, 22.5°, 45°, 67.5°, and 90°, respectively, and the maximum strain was 0.17 for all orientation angles. In particular, the simulated tensile strength values measured were higher than the experimental values at all orientation angles. Conversely, the maximum strain values predicted by the simulation were significantly lower at all orientation angles. Simulation and experimental results indicated that both composites had the highest strength at 90°. This can be explained because 90° alignment provides the highest reinforcement, as the fibers are oriented in the direction of the applied load [32,33]. According to Shi et al., such orientation effects might be attributable to the fact that various angles of fibers provide a variable level of resistance to rotation [24].

**Figure 5:**
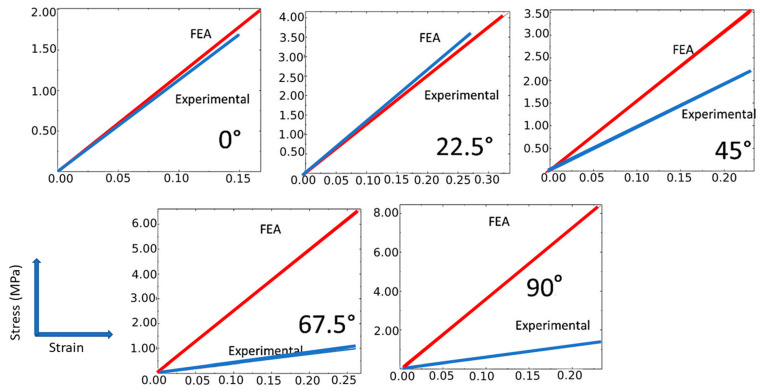
Stress–strain response of nylon/Kevlar fiber composites with different orientations.

The stress–strain profiles for different nylon/Kevlar fiber composites with different orientation angles are shown in Figure 5. The stress–strain relationship was linear in all composites. The maximum stress was 1.7 MPa, 3.62 MPa, 2.2 MPa, 1.0 MPa, and 1.4 MPa for the orientation angles of 0°, 22.5°, 45°, 67.5°, and 90°, respectively, and the maximum strain was 0.15, 0.27, 0.25, 0.25, and 0.23 for the corresponding orientation angles. Consistent with the findings of nylon/glass fiber composites, in nylon/Kevlar composites, the deviation between the experimental and simulated profiles varied depending on the orientation angle. It was found to be highest at 90° and lowest at 0°. In the simulated profiles, the maximum stress was 2.0 MPa, 4.13 MPa, 3.54 MPa, 6.58 MPa, and 8.4 MPa for the orientation angles of 0°, 22.5°, 45°, 67.5°, and 90°, respectively. The maximum strain was 0.16, 0.33, 0.23, 0.27, and 0.23 for the orientation angles of 0°, 22.5°, 45°, 67.5°, and 90°, respectively. In the simulated profiles, the maximum tensile strength was observed at 90°, while in the experimental profiles, the maximum strength was obtained at 22.5°. These results indicate that factors other than orientation determine the micromechanics of 3D-printed composites. The most notable among these could be interfacial adhesion, internal stress, nucleation, and viscoelasticity [31,34,35].

**Figure 6:**
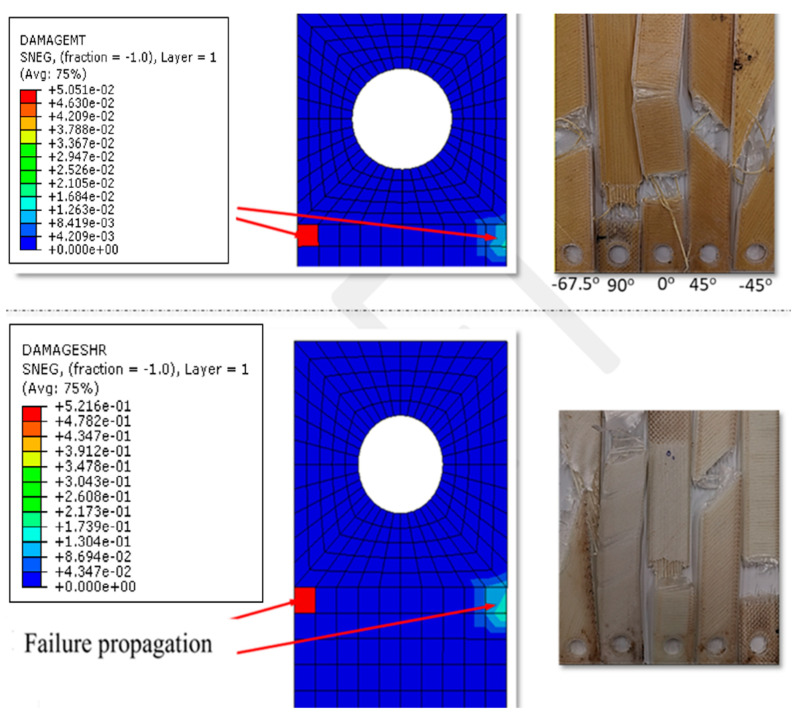
Failure of composite layup predicted by FEA and photographs of a test specimen after the mechanical test (nylon/Kevlar composites (**top**) and nylon/glass fiber composites (**bottom**)); the failure samples had dimensions of 150 mm × 25 mm × 2.5 mm (L × W × H).

To further understand the micromechanics of the nylon/Kevlar composites, the damage parameters were recorded for different failure modes (Figure 6). The progressive failure criterion is described by five components (DAMAGEFT, tensile fiber damage; DAMAGEFC, compressive fiber damage; DAMAGEMT, tensile matrix damage; DAMAGEMC, compressive matrix damage; and DAMAGESHR, shear damage).

The criterion is fulfilled when the value of both of the damage evolution parameters is in the range, which means only the percentage loss of initial stiffness of the structure, while after reaching the value of 1, there is a 100% loss of stiffness. These parameters range from 0 (corresponding to no damage) to 1.0 (corresponding to complete damage). This presents the progressive damage predictions for four different failure modes for the Kevlar composites. The results indicate that the damage is mediated by matrix tensile damage (i.e., DAMAGEMT) and fiber tensile damage (DAMAGEFT) in all layers. This compression failure mechanism was also evident in some layers (Nylon: −45° and Kevlar: 90°). Our results corroborate Muflikhun’s conclusion that the orientation and infill density might impact the quality and efficiency of a product manufactured using a 3D printing fabrication technique [36]. The FEA findings indicate that failure propagation begins near the hole, as shown in Figure 6. Rakesh et al. used FEA to predict failure in polymer laminates with a drilled hole [37]. Their simulation results also indicated that the initial damage occurred around the drilled hole, and glass fiber–matrix debonding also played an important role. In this study, fiber–matrix debonding was also evident in all samples. However, it may be noted that in the experimental results, the location of the failure point varied depending on the orientation angle. These results indicate that in 3D-printed structures, the hole is not always the failure site under uniaxial stress. Furthermore, unlike conventional drilling techniques involving structural damage, in 3D printing samples, such damage is not anticipated [38,39,40,41]. Moreover, 3D printing technology needs to be improved, particularly in the context of enhancing interfacial compatibility between different types of fibers and polymer matrices and maintaining a precise orientation level [42]. Plasma treatment of fibers [43], interfacial grafting [44], and tailoring fiber orientations [45] are the critical approaches that can be employed to enhance the interfacial interactions between polymer and fiber during 3D printing.

## 4. Conclusions

3D printing is used for rapid prototyping and to create objects with complex geometries that would otherwise be difficult to produce using traditional manufacturing methods. In this study, 3D-printed, fiber-reinforced composites with a hole were fabricated, and the influence of the fiber orientation on the stress–strain profile was investigated experimentally and numerically. The stress–strain relationship was linear in all composites, and Kevlar-based composites exhibited mechanical strength superior to glass-based composites. The results provided critical insights into the mechanical failure of fiber-reinforced, 3D-printed structures. The highest stress levels for Kevlar-based composites were 1.7 MPa, 3.62 MPa, 2.2 MPa, 1.0 MPa, and 1.4 MPa for the orientation angles of 0°, 22.5°, 45°, 67.5°, and 90°, respectively. The nylon/Kevlar composites had higher strength than the nylon/glass fibers. In the nylon matrix, fiber orientation significantly impacted the final mechanical properties. The simulation findings projected that failure propagation would begin in locations nearer to the hole. The damage behavior of the composite layout was represented by the Hashin damage theory and the FEA technique. However, the final results indicated a substantial difference between the simulated and experimental values, which varied according to the fiber orientation. These findings highlight the need for significant advancements in 3D printing methods to achieve the full potential of fiber-reinforced, 3D-printed objects. Designers and engineers should pay close attention to fiber orientation and employ a geometry appropriate for specific load-bearing applications. Further studies related to fiber–matrix interactions are necessary to enhance predictive accuracy. It should also be emphasized that, in practical applications, 3D-printed objects will be subjected to multiple stresses, such as bending, torsion, out-of-plane, and compression; further studies should be conducted on different stresses as applicable to the final application conditions.

## Figures and Tables

**Table 1:** Adopted elastic material properties of the printed materials.

Material Properties	Kevlar	Glass	Nylon
Longitudinal elastic modulus-E1 (MPa)	29998	24998	378
Transverse elastic modulus-E2 (MPa)	9998	4998	378
In-plane shear modulus-G12 and G23 (MPa)	4998	998	142
Poisson’s ratio- $\nu_{12}$	0.21	0.21	0.34
Axial strength in tension-$f_{1T}$ (MPa)	598	558	54
Axial strength in compression-$f_{1c}$ (MPa)	478	446	43
Transverse strength in tension-$f_{2T}$ (MPa)	598	558	30
Transverse strength in compression-$f_{2c}$ (MPa)	478	446	10.2
Shear strength in tension-$f_{1s}$ (MPa)	38	38	7.2
Shear strength in compression-$f_{2s}$ (MPa)	38	38	11

## Data Availability

All the raw data supporting the conclusion of this paper were provided by the author.
